# An Evaluation of Turkish Dentists’ Approach to Indirect Pulp Capping and Material Preferences: A Questionnaire-Based Survey

**DOI:** 10.3390/medicina61071120

**Published:** 2025-06-20

**Authors:** Baturalp Arslan, Batu Can Yaman, Özge Çeliksöz, Havva Can Aydın

**Affiliations:** Department of Restorative Dentistry, Faculty of Dentistry, Eskisehir Osmangazi University, 26040 Eskisehir, Turkey; batucanyaman@hotmail.com (B.C.Y.); ozgeeozdil@gmail.com (Ö.Ç.); havvacan268@gmail.com (H.C.A.)

**Keywords:** dentistry, dental pulp capping, surveys and questionnaires

## Abstract

*Background and Objectives*: The aim of this study was to evaluate how the indirect pulp capping treatment approaches and material choices used by dentists actively practicing in Turkey vary according to demographic data. *Materials and Methods*: Dentists practicing in Turkey were included in this study. A 13-question survey was used and distributed to the participants via social media. The statistical analysis of the data obtained from this study was performed using IBM SPSS v23. The chi-square test was used to compare categorical variables between groups, and multiple comparisons of the proportions were analyzed using the Bonferroni correction. The results of the analysis are presented as frequencies (percentages) for categorical data. The significance level was set at *p* < 0.05. *Results*: A total of 402 dentists from across Turkey participated in this study. A total of 331 participants (82.3%) reported that they performed indirect pulp capping treatment. The most commonly used materials for indirect pulp capping were Ca(OH)_2_;-containing liners (73.4%) and glass ionomer cement (58.3%). The use of amalgam and cotton roll isolation was more common among dentists working in the public sector, whereas rubber dam isolation and the use of contemporary materials such as MTA and Biodentine were more frequently observed among dentists working in the private sector. *Conclusions*: Significant differences were found in the dentists’ indirect pulp capping approaches and the materials they used based on their specialty, years of experience, and workplace setting. These findings suggest that dentists’ knowledge and experience regarding indirect pulp capping should be enhanced during their education and post-graduation training.

## 1. Introduction

One of the greatest challenges in modern restorative dentistry is achieving remineralization of hypomineralized carious dentin and preserving pulp viability. In the conventional caries tissue removal protocols, accidental opening of the pulp can occur during the complete and non-selective removal of caries tissue. This is often referred to as “overtreatment” and can lead to unnecessary damage to the pulp [1]. Furthermore, it has been observed that even when caries is thought to be completely removed, 25 to 50% of bacteria continue to colonize the cavity. Therefore, modern opinion and the available studies recommend the intelligent management of the structure and quantity of bacterial biofilm during caries cleaning protocols, rather than complete removal of the bacteria [2].

Preventing pulp exposure in asymptomatic deep dentin caries cases allows the pulp to be preserved, avoiding the need for invasive endodontic procedures [3]. Therefore, minimally invasive treatment strategies based on biological healing and selective caries removal are recommended for managing deep carious lesions. This approach not only helps to maintain pulp health but also prevents unnecessary loss of the dental tissue [4].

With the development of the materials used in restorative dental treatment and a better understanding of pulp biology, vital pulp therapy is becoming an increasingly preferred treatment. In the past, these treatments were limited to teeth with incomplete root development due to their unpredictable outcomes; however, their indications have significantly expanded today [5]. A critical factor affecting the success of vital pulp therapies is an accurate assessment of pulpal health. Although histological examination is the “gold standard” for determining pulp vitality, clinicians rely on clinical signs and radiographic findings in practice. What matters most is the ability of the pulpal cells to initiate reparative dentin formation and halt the progression of caries [6].

Calcium hydroxide (Ca(OH)_2_) has been accepted as the long-term gold standard in vital pulp treatments. Ca(OH)_2_ has a long history of success in the literature, with antibacterial efficacy thanks to its dentin-forming capacity and high pH value. However, the disadvantages of Ca(OH)_2_, such as its dissolution over time, lack of high coverage, and microleakage, have resulted in the search for new materials [7]. Accordingly, mineral trioxide aggregate (MTA), a calcium-silicate-based radiopaque material, was introduced into the market. Thanks to its biocompatibility and effective sealing properties, MTA has shown success in many areas, from use as a retrograde filling material to use as a pulp capping agent. Compared to the dentin bridge formed by Ca(OH)_2_, it has been reported to form a dentin bridge with fewer pores and higher quality. However, MTA also comprises disadvantages, such as difficulty in handling, long hardening times, discoloration, and high costs [8]. Recently, a new generation of fast-setting calcium-silicate-based biomaterials have been developed that harden in a shorter time and have less potential for discoloration compared to conventional MTA. One of these relatively new biomaterials is Biodentine (Septodont, Saint Maur des Fosses, France), introduced in 2011. It is easy to apply thanks to its capsule form, allows for single-session restorations, and does not contain bismuth oxide, which causes discoloration [9].

Other materials developed in recent years to overcome the disadvantages of MTA are light-curing calcium silicate materials. One of these materials, Theracal LC (Bisco, Schamurg, IL, USA), contains polymerizable methacrylate monomers, light-cures, and allows the restoration to be completed in a single session during a vital pulp therapy procedure. The manufacturer states that Theracal LC can be used as a liner under class I-II restorations (composites, amalgams, glass ionomer cements) as a direct and indirect pulp capping agent [10].

Indirect pulp capping is an approach within the scope of vital pulp therapy. It is based on the removal of non-mineralized dentin tissue during caries cleaning, leaving the deepest and most intact thin layer of dentin in the carious cavity and sealing the affected dentin with a biocompatible material to create a seal [3]. During the implementation of this procedure, the material used is of great importance to the preservation of tooth vitality and the success of the treatment [11]. Indirect pulp capping is widely used in dentistry. There are some differences in dentists’ use of the current materials used during indirect capping and their application of the treatment protocols. It is of great importance for indirect pulp capping, which is one of the most common treatments in today’s dental clinical practice, to be applied with the right materials. Therefore, it should be evaluated whether dentists follow the current literature. The aim of this web-based questionnaire study was to assess whether dentists’ specialties and practice locations influenced their indirect pulp capping practices and material preferences.

The null hypothesis was that dentists’ specialties affects the way in which they apply indirect pulp capping and their material preferences. The second null hypothesis was that dentists’ place of practice affects the way in which they apply indirect pulp capping and their material preferences.

## 2. Materials and Methods

The ethics committee approval required for this study was obtained from Eskisehir Osmangazi University Non-Interventional Clinical Research Ethics Committee, Turkey (Decision Date: 14.12.2021; Decision No: 18). Dentists who voluntarily agreed to participate were included, and this study was conducted in accordance with the Declaration of Helsinki. Before proceeding to the questionnaire form, the participants were presented with an “Informed Consent Form”, in which the purpose of this study was explained in detail. Data collected from participants who agreed were included in this study. The aim of this study was to evaluate the awareness and knowledge levels of dentists working in the public and private sectors in Turkey of the pulp capping treatment approaches and the materials they used, together with the demographic data of the dentists.

The questionnaire used in this study was conducted using “Survey Google Forms” (via docs.google.com/forms). In order to finalize the questions in the questionnaire, a pilot study was conducted on a group of ten persons working in the Department of Dentistry at Eskisehir Osmangazi University. The current curriculum in the field of restorative dental treatment was taken into consideration, and the survey questions were formulated accordingly. The sample size required for this study was calculated as a minimum of 194 participants according to a power analysis G*Power (version 3.1.9.7, Heinrich Heine Universität Düsseldorf) (effect size 0.3, bias level 5% and power level 90%). The survey was conducted between January 2022 and December 2022 and included dentists and dental specialists working in private or public institutions in Turkey.

### 2.1. The Survey

A questionnaire form was created for the evaluation of the indirect pulp capping treatment approaches and materials used among dentists by evaluating questions from similar studies in the literature [12,13].

**Figure 1:**
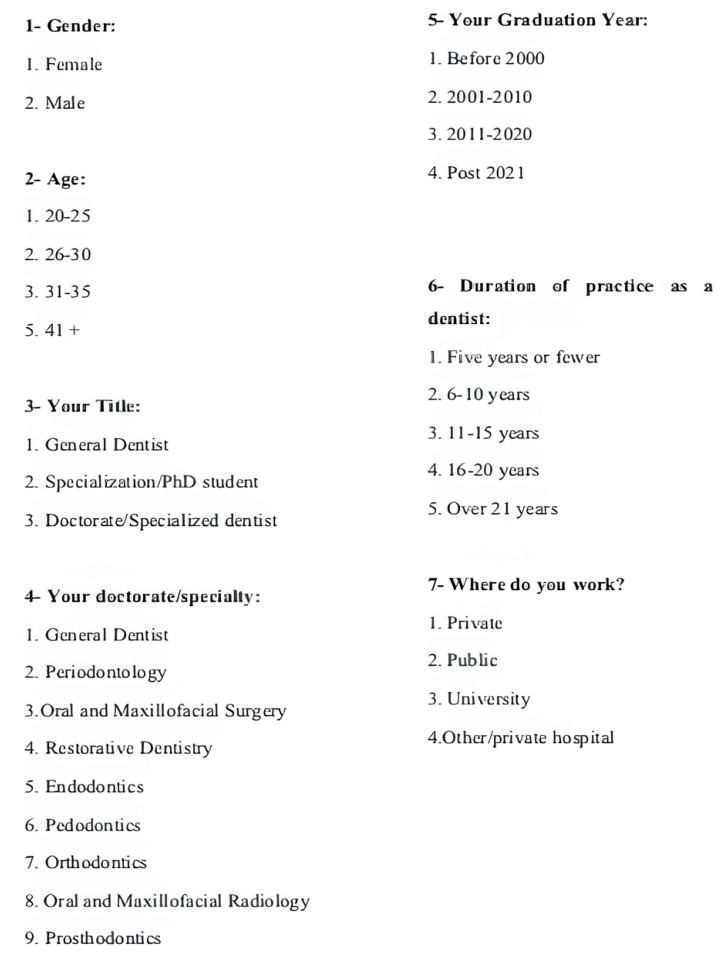
Questionnaire questions related to demographic data.

**Figure 2:**
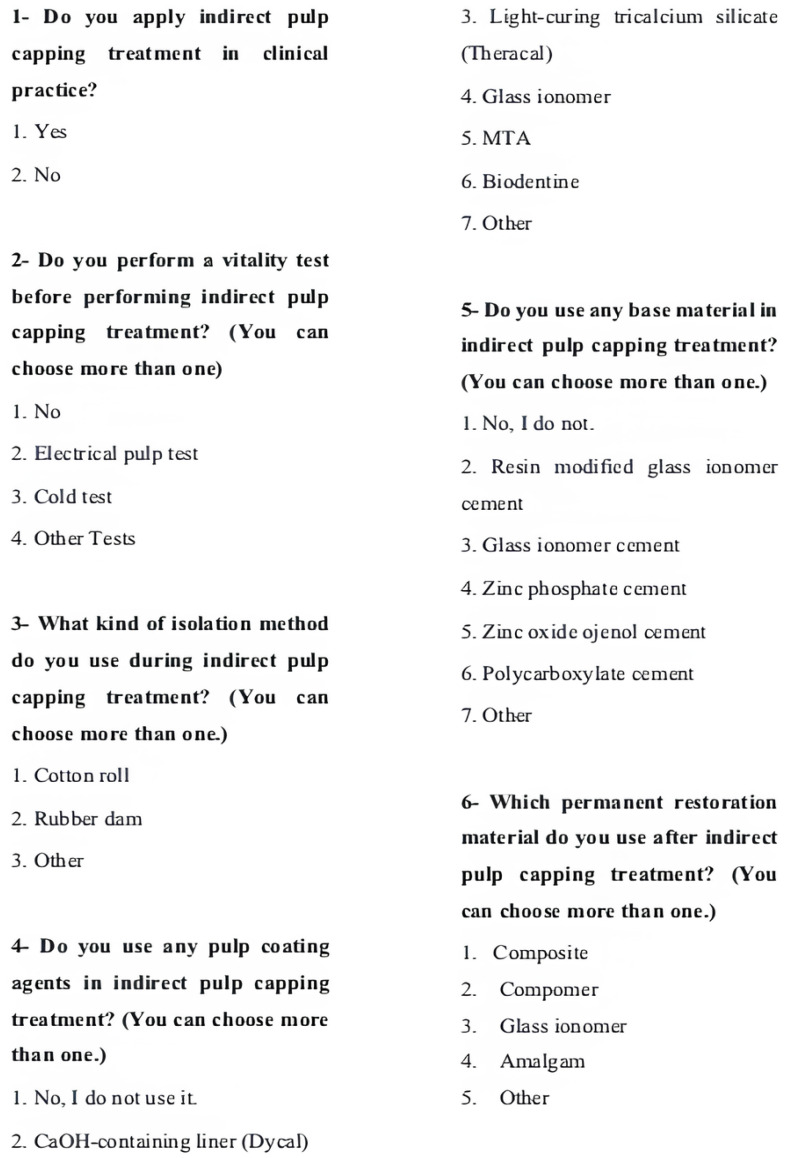
Questionnaire questions about indirect pulp capping.

The questionnaire consisted of thirteen questions in total and had two sections: (1) the demographic information of the participants, such as gender, age, and duration of dental practice (Figure 1), and (2) questions about whether they applied indirect pulp capping treatment and the materials preferred by those who applied indirect pulp capping treatment (Figure 2). The questionnaire was made available to 1500 dentists through social media, and the survey link was sent. Data obtained from participants who gave incomplete answers and those who did not want their answers to be used for scientific purposes were excluded from the evaluation, and questionnaires from 402 participants were included in this study. IP protection protocols were used to prevent the respondents from logging in and answering more than once via social media. Participants were able to provide information about all of the clinical procedures they performed, as they could insert more than one mark in the multiple-choice questions in the questionnaire. The data collected from the participants were transcribed into a MS Excel spreadsheet with the help of Google Surveys.

### 2.2. Inclusion Criteria

Physicians with or without dental specialization working in universities and private or public hospitals participated this the study.

### 2.3. Exclusion Criteria

Student interns and physicians who had completed their education in educational institutions outside of the borders of the Republic of Turkey were not included in this study.

### 2.4. The Statistical Analysis

The statistical analysis of the data collected in this study was performed using IBM SPSS v23 (IBM Corp., Armonk, NY, USA). The chi-square test was used to compare the responses among dentists, and multiple comparisons of the proportions were made using the Bonferroni correction. The significance level of the analyses was set at *p* < 0.05.

## 3. Results

A total of 402 dentists participated in the survey. Of the participants, 266 (66.2%) were female and 136 (33.8%) were male; 55.2% were dentists; 30.6% were specialization/PhD students; and 14.2% were doctorate/specialized dentists. The highest number of participants was between 26 and 30 years of age, while 331 (82.3%) participants applied indirect pulp capping treatments, and 71 (17.7%) participants stated that they did not. The participants were allowed to give multiple answers to questions other than those about demographic data.

A difference was found between the answers given to the question “Do you apply indirect pulp capping treatment in clinical practice?” according to gender (*p* = 0.027), with 85.3% of the female respondents and 76.5% of the male respondents answering “yes”. According to title, the most common practitioners were doctorate/specialized dentists, with a rate of 87.7% (Table 1).

Although 71.1% of the dentists applied the treatment, they did not perform any vitality tests. However, this rate was 63.9% in dentists working in the field of restorative dentistry. In contrast, 36.1% of the restorative dentistry specialists said that they performed electric pulp tests. The use of cold testing (45.8%) and electrical pulp testing (EPT) (50%) was common among endodontic specialists (Table 2).

According to place of employment, 98.9% of the physicians working in the public sector preferred isolation with cotton rolls. The frequency of rubber dam use was highest among university physicians, with a rate of 31% (Table 3).

**Figure 3:**
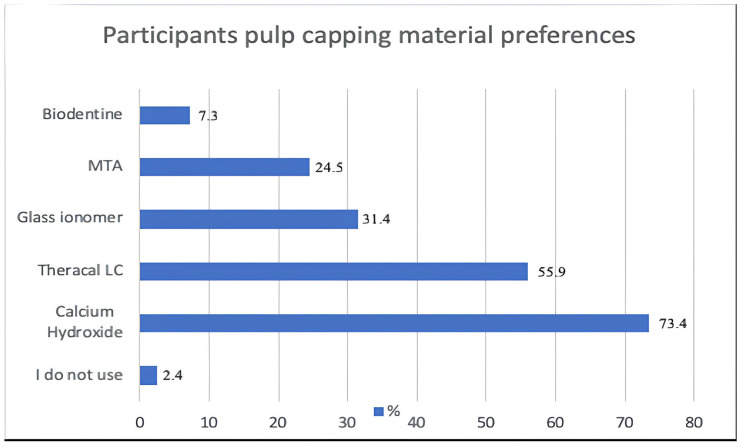
Participants’ pulp capping material preferences.

In this study, the most commonly used pulp capping material in indirect pulp capping treatment was determined to be Ca(OH)_2_-containing liners, with a rate of 73.4% (Figure 3).

The use of Theracal LC was most common among restorative dentistry specialists, with a rate of 80.6%. Since there was only one orthodontic physician practicing the treatment, this response was not significant. In preference for the use of MTA, it was seen that physicians working in the field of pedodontics were at the forefront. The most preferred pulp capping agent by the dentists was Ca(OH)_2_, at 75.3% (Table 4).

When the pulp capping agent usage preferences were analyzed according to place of work, it was observed that the most preferred agent in the public sector was Ca(OH)_2_, at 85.4%. It was determined that the group that most preferred the use of MTA material, at 29.4%, was that of physicians working at a university (Table 5).

**Figure 4:**
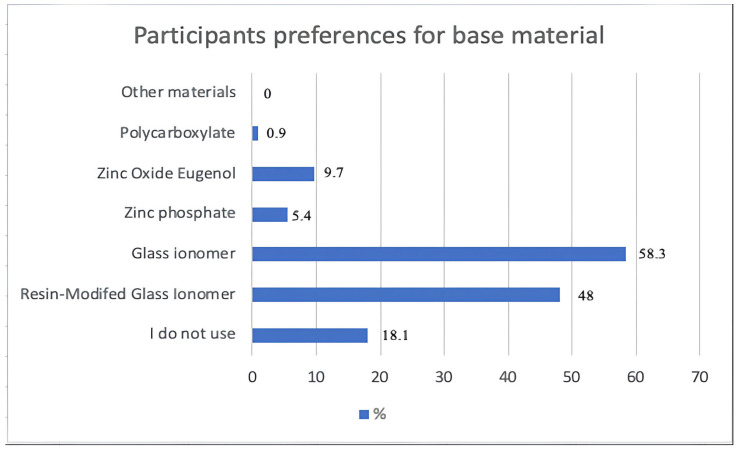
Participants’ base material preferences.

In this study, glass ionomer cement was the most commonly used base material in indirect pulp capping treatment, with a rate of 58.3% (Figure 4).

When analyzed according to place of work, it was observed that the group that most preferred the use of compomer as permanent restorative material was that of physicians working at a university. It was determined that amalgam use was very common among physicians working in the public sector (50.6%). None of the physicians working in private practice preferred amalgam as a permanent restorative material (Table 6).

## 4. Discussion

In teeth with deep dentin caries, the conditions affecting the tooth and oral environment, such as the inflammation status of the pulp, the repair ability, the exposure to caries, and the isolation conditions, can subjectively affect the treatment and material choices for dentists [14]. The aim of this study was to measure dentists’ knowledge about the application of indirect pulp capping treatment and determine the correct indication and the results of the treatment. For this purpose, 402 dentists working in different institutions throughout Turkey participated in this study. While 331 (82.3%) participants applied indirect pulp capping treatment, 71 (17.7%) participants stated that they did not. The two hypotheses of this study were accepted; the specialties and working locations of the dentists were found to affect the way they applied indirect pulp sealants and their preferences in the material they used.

Today, successful vital pulp therapy can be performed using the current restorative materials. However, it should be known that the treatment may not be successful in every case. In this study, when the rate of indirect pulp capping application was examined according to specialty, the highest rate was found among restorative dentistry specialists, at 91.2%. In a survey study conducted among dentists in China, it was observed that endodontists preferred to perform vital pulp therapy more frequently compared to general practitioners. This result shows that post-graduate education in dentistry plays an important role in determining the treatment options [15].

There are many factors to consider when deciding on the treatment options for vital pulp therapy. Following advances in dentistry, more invasive treatment options are being replaced by conservative options. However, a better understanding of pulp biology is required before deciding on an approach that requires the preservation of pulp vitality [16]. In this study, the participants were asked whether they performed a vitality test before pulp capping treatment. It was determined that 67.3% of the participants did not perform a vitality test in indirect pulp capping treatment. Electrical pulp testing is used to differentiate reversible pulpitis from irreversible pulpitis; however, it does not provide accurate information about pulp inflammation, and positive responses may be encountered in necrotic teeth [17]. Therefore, it is not sufficient for determining the vitality of the tooth alone. When the vitality methods were analyzed according to place of employment, the highest rate (85.4%) of not performing vitality tests in indirect pulp capping was seen among public sector employees. We think that the insufficient time that can be allocated to each patient in the public sector may lead to this result. Apart from this, not a single respondent used current methods such as pulse oximetry or laser Doppler flowmetry, which are the most accurate methods for determining vitality. In a survey conducted in the United Kingdom, dentists from different sectors were asked about their approach to the treatment of deep caries. They were asked which methods they used for diagnosis, and the highest percentage (96.1%) reported that they made decisions based on preoperative symptoms, whereas 50.2% reported using cold testing and 15.6% used electrical pulp testing [18]. The low rate of vitality testing was similar to that in the present study.

Rubber dam isolation in restorative and endodontic treatments is the most ideal method for infection control and the long-term success of the restoration [19]. However, there is still widespread reluctance among dentists to use rubber dams [20]. In a questionnaire study evaluating the approach of Turkish dentists to deep caries lesions, it was observed that the use of rubber dams was very low, which supports the data from our study. Despite its many advantages, we attribute the low frequency of rubber dam use to the limited time allocated to each patient, especially in public hospitals [21]. In this study, 21.5% of the participants stated that they used a rubber dam in indirect pulp capping treatment. When analyzed by specialty, 100% of the orthodontic specialists stated that they used a rubber dam. However, only one participant checked this option, and nine participants were excluded from the evaluation because they stated that they did not apply the treatment. Apart from this, the most frequent users of rubber dams according to specialty were endodontic specialists, with 54.2% using these in indirect pulp capping treatment. This may be attributed to the fact that rubber dam use is more common among endodontists in Turkey. In a study conducted in India, the frequency of rubber dam use by endodontists during root canal treatment was examined, and it was determined that 52% used them occasionally and 34% used them continuously [22]. In a survey study conducted in Tianjin, the frequency of rubber dam use was examined, and it was concluded that 63.3% used rubber dams. However, the rate of those who always used one in the restoration of deep caries was 0.4% [23]. In another study conducted in England, 30.3% of physicians stated that they always used rubber dams, while 37.4% stated that they used them in some cases [24].

We enquired about pulp capping agent use in this study, and multiple-answer options were presented. As a result, the most commonly used material was found to be Ca(OH)_2_. In a study conducted in England, Ca(OH)_2_ was also the most preferred material [25], as it was in a study conducted in Brazil, and in line with this study, it was preferred by 80.3% in the indirect pulp capping process [13]. In this study, the second most frequently used material in indirect pulp capping treatment was determined to be Theracal LC (55.9%).

In a large-scale study conducted in a number of countries in Europe, a questionnaire survey was conducted to determine which pulp capping materials were taught about in schools. The most popular material for indirect pulp capping was determined to be Ca(OH)_2_ [26]. We suspect that the main reason for the unpopularity of MTA in our country is that it is not an economical material, as well as the possibility that there is insufficient knowledge and experience in undergraduate education. As a result of another study examining the adaptation of MTA, Biodentine, and Theracal LC materials to the dentin surface, the importance of removing economic limitations by ensuring affordability was mentioned [27]. Biodentine, a current pulp capping agent developed as an alternative to MTA, is a bioactive material similar to dentin and has been reported to accelerate healing and dentin bridge formation on contact with the pulp [28,29]. In this study, Biodentine, which is an expensive material, was mostly preferred by endodontic specialists, at 35%. In a survey study conducted in Karnataka, the materials used among pediatric dentists during capping treatment for deciduous and young permanent teeth were examined, and the most preferred material was Biodentine (43%) [30]. When the pulp capping agent used in indirect pulp capping treatment was examined according to place of work, the material most preferred by the public sector dentists was Ca(OH)_2_, with a rate of 85.4%. Those working in other/private hospitals answered “Theracal LC” at a rate of 75.8% and were the group that used this material most frequently.

Base material use was enquired about in this study, and glass ionomer cement was the most preferred material in indirect pulp capping treatment. Resin-modified glass ionomer cement, which was produced to improve the physical properties of traditional glass ionomer cement, has started to be used in clinical practice thanks to its advantages such as its lengthy effective period and easy application [31]. In another study, this material, which was successful in indirect pulp capping treatment, caused an inflammatory response in direct pulp capping treatment and showed more cytotoxic effects than those of traditional glass ionomer [32].

Regarding the choice of base material according to place of employment, public sector employees most prominently used zinc oxide eugenol in indirect pulp capping (25.8%). Zinc oxide eugenol is a therapeutic material with both anti-inflammatory and analgesic effects. However, the release of eugenol may have a cytotoxic effect and reduce the success of the treatment [33]. If zinc oxide eugenol cement is used with composite resin material, glass ionomer cement should be placed in the interlayer because it adversely affects the polymerization of the resin [34]. In a survey study conducted in Brazil, the restoration of deep caries lesions was investigated, and 69.2% of the participants stated that they applied Ca(OH)_2_, glass ionomer cement, and composite restoration together, and 16.3% of the participants stated that they used glass ionomer cement and a composite together, while 1.1% used only a composite [13].

In our study, the participants were asked about the permanent restorative material that they used and were given multiple-answer options. Composites were the most preferred restorative material in both indirect pulp capping (97.6%) and direct pulp capping (95.7%). However, their other restorative material preferences also varied according to both specialty and place of work.

When analyzed according to specialty, it was determined that pedodontists (28.1%) preferred the use of compomers in indirect pulp capping treatment the most. We attribute the preference for compomers among pediatric dentists to the fact that it is much more difficult to achieve cooperation in pediatric patients. In a survey study conducted among dentists and dental students in Russia, minimally invasive dentistry approaches to occlusal and proximal caries lesions and the materials used were inquired about; composites were the most preferred material in both groups, followed by composites and glass ionomer cement together. When the preference for amalgam was examined, it was reported that none of the dental students preferred amalgam, and only three dentists preferred amalgam for occlusal caries lesions, which is consistent with this study [35].

When permanent restorative material preferences were analyzed according to place of employment, 50.6% of the public sector employees preferred amalgam use in indirect pulp capping treatment. We attribute the higher preference for amalgam among the public sector employees to the fact that it is both less costly than composites and reduces the time spent at a patient’s bedside.

In a survey study conducted in Turkey on deciduous tooth treatment, material preferences were inquired about, and the most preferred permanent restorative materials were compomers, glass ionomer cement, composites, and amalgam, respectively. Similar to this study, the preference for amalgam was found to be higher in public sector employees than in private sector employees [12]. In a survey study on amalgam preference conducted among dentists and dental students in Arabia, 80.7% of the participants reported that they did not prefer amalgam in clinical practice. The reason for not preferring amalgam was a lack of aesthetics, with a high rate of 77.1%. However, 72.3% of the participants stated that a good amalgam restoration does not need to be replaced with amalgam or composites. In this study, similar to the previous one, employees working in the private sector preferred amalgam less [36]. In a comprehensive 13-year study conducted in a dental school in Israel, the distribution of the permanent restorative materials used in the student clinic was examined according to year. It was concluded that composites were preferred more frequently than amalgam by the end of these 13 years. At the end of this period, the preference for composites increased from 54.7% to 81.9% in one-facet restorations; from 33.3% to 64.3% in two-facet restorations; and from 27.92% to 48.66% in three-facet restorations [37]. Both in this study and in other studies around the world, composites have replaced amalgam. Considering that the aesthetic expectations of patients are an important parameter in directing the treatment today, it is understandable that physicians are turning towards composites, which are more aesthetically pleasing materials.

The survey study, which was conducted in a large population for a long period of time, provided qualified results supporting the hypotheses. The materials used varied greatly depending on place of work and specialty. This study has once again made us aware that access to MTA and Biodentine, which are among the current materials, is very difficult, especially in public hospitals. This study will serve as a guide for future studies that will evaluate whether there will be any change in the current material preferences or increases in the frequency of rubber dam use. However, since the survey was conducted through social media, the inability to reach dentists who do not actively use social media is one limitation of this study. Another limitation is that all of the procedures for the diagnosis and treatment of advanced caries lesions were not included in the survey study. In order to validate the data obtained in the survey, it should be administered in larger and more diverse populations. Further studies are needed to improve the current knowledge in the literature.

## 5. Conclusions

In this large-scale study, the indirect pulp capping treatment approaches and materials used among dentists and dental specialists in Turkey were evaluated according to their specialty and practice location. It was concluded that the majority of the dentists did not perform vitality testing. It was determined that the frequency of rubber dam use was higher in physicians working at universities or in physicians who were new to the profession, but this was not sufficient. When the materials used were evaluated, it was observed that physicians working in the public sector preferred the old materials that they were used to, instead of the current materials. In terms of their pulp capping agent preferences, it was determined that the preference for MTA material was lower, compared to that seen in other studies conducted worldwide. When the base materials used were analyzed, the most preferred material was glass ionomer cement, and similar results to those from other studies were obtained. When the permanent restoration material used was analyzed, the most preferred material was composites, and here again, similar results to those from other studies were acquired. According to the results we obtained from this study, similar investigations should be repeated more frequently, and this information should be imparted, encouraging physicians to follow the literature. In addition, continuing educational modules on vital pulp treatment are recommended. Future studies should include other procedures for the diagnosis and treatment of advanced caries lesions and should be conducted in larger and more diverse populations to confirm the data obtained in this study.

## Figures and Tables

**Table 1:** Distribution of the answers to the question “Do you apply indirect pulp capping treatment?” according to demographic characteristics.

		Do You Use Indirect Pulp Capping in Clinical Practice?		*p*
		Yes	No	
		*n* (%)	*n* (%)	
Gender	Female	227 (85.3)	39 (14.7)	**0.027**
	Male	104 (76.5)	32 (23.5)	
Age	20–25	72 (83.7)	14 (16.3)	0.211
	26–30	172 (82.3)	37 (17.7)	
	31–35	51 (89.5)	6 (10.5)	
	36–40	14 (73.7)	5 (26.3)	
	41+	22 (71)	9 (29)	
Title	General dentist	190 (85.6) _a_	32 (14.4)	**0.013**
	Specialization/PhD student	91 (74)	32 (26)	
	Doctorate/specialized dentist	50 (87.7) _ab_	7 (12.3)	
PhD/specialization branch	General dentistry	190 (86.4) _a_	30 (13.6) _a_	**<0.001**
	Periodontology	3 (33.3) _bc_	6 (66.7) _bc_	
	Oral and maxillofacial surgery	4 (26.7) _bc_	11 (73.3) _bc_	
	Restorative dentistry	62 (91.2) _a_	6 (8.8)	
	Endodontics	24 (100)	0 (0)	
	Pedodontics	32 (100)	0 (0)	
	Orthodontics	1 (10)	9 (90)	
	Prosthodontics	15 (68.2) _ac_	7 (31.8) _ac_	
Graduation year	Before 2000	14 (77.8)	4 (22.2)	0.759
	2001–2010	27 (77.1)	8 (22.9)	
	2011–2020	218 (83.5)	43 (16.5)	
	Post 2021	72 (81.8)	16 (18.2)	
Years of professional experience	5 years or fewer	223 (83.5)	44 (16.5)	0.110
	6–10 years	68 (86.1)	11 (13.9)	
	11–15 years	17 (77.3)	5 (22.7)	
	16–20 years	14 (73.7)	5 (26.3)	
	Over 21 years	9 (60)	6 (40)	
Employed institution	Private	48 (92.3)	4 (7.7)	0.051
	Public	89 (81.7)	20 (18.3)	
	University	126 (76.4)	39 (23.6)	
	Other/private hospital	66 (89.2)	8 (10.8)	

Chi-square test, a–c: there is no difference between groups with the same letter in each row; *n*: frequency; %: percentage; *p* < 0.05: There is a statistically significant difference.

**Table 2:** The distribution of answers to the question “Do you perform a vitality test before applying indirect pulp capping treatment?” according to specialty.

		General Dentist	Periodontology	Oral and Maxillofacial Surgery	Restorative Dentistry	Endodontics	Pedodontics	Orthodontics	Prosthodontics	*p*
		***n* (%)**	***n* (%)**	***n* (%)**	***n* (%)**	***n* (%)**	***n* (%)**	***n* (%)**	***n* (%)**	**<0.001**
Do you perform a vitality test before performing indirect pulp capping treatment?	No	135 (71.1) _a_	3 (100)	2 (50) _ab_	39 (63.9) _a_	6 (25) _b_	23 (71.9) _a_	1 (100)	13 (86.7) _a_	
	Electrical pulp test	25 (13.2) _a_	0 (0)	2 (50) _ab_	22 (36.1) _bd_	12 (50) _bc_	4 (12.5) _ad_	0 (0)	1 (6.7) _ab_	
	Cold test	42 (22.1)	0 (0)	1 (25)	10 (16.4)	11 (45.8)	9 (28.1)	0 (0)	2 (13.3)	
	Other tests	2 (1.1) _a_	0 (0)	0 (0)	1 (1.6) _ab_	3 (12.5) _b_	0 (0)	0 (0)	0 (0)	

Chi-square test, a–d there is no difference between groups with the same letter in each row; *n*: frequency; %: percentage; multiple responses; oral and maxillofacial radiology is not included in this comparison; *p* < 0.05: There is a statistically significant difference.

**Table 3:** The distribution of answers to the question “What kind of isolation method do you use during indirect pulp capping treatment?” according to place of employment.

		Private	Public	University	Other/Private Hospitals	*p*
		***n* (%)**	***n* (%)**	***n* (%)**	***n* (%)**	**<0.001**
What kind of isolation method do you use during indirect pulp capping treatment?	Cotton roll	41 (85.4) _a_	88 (98.9) _b_	113 (89.7) _a_	57 (86.4) _a_	
	Rubber dam	11 (22.9) _a_	3 (3.4) _b_	39 (31) _a_	18 (27.3) _a_	
	Other	0 (0)	0 (0)	1 (0.8)	1 (1.5)	

Chi-square test, a, b: there is no difference between groups with the same letter in each row; *n*: frequency; %: percentage; multiple responses; *p* < 0.05: There is a statistically significant difference.

**Table 4:** The distribution of answers to the question “Do you use any pulp coating agents in indirect pulp capping treatment?” according to specialty.

		General dentist	Periodontology	Oral and Maxillofacial Surgery	Restorative Dentistry	Endodontics	Pedodontics	Orthodontics	Prosthodontics	*p*
		***n* (%)**	***n* (%)**	***n* (%)**	***n* (%)**	***n* (%)**	***n* (%)**	***n* (%)**	***n* (%)**	**<0.001**
Do you use any pulp coating agents in indirect pulp capping treatment?	No	4 (2.1)	0 (0)	0 (0)	1 (1.6)	2 (8.3)	1 (3.1)	0 (0)	0 (0)	
	Ca(OH)_2_	143 (75.3)	3 (100)	4 (100)	41 (66.1)	16 (66.7)	24 (75)	0 (0)	12 (80)	
	Theracal LC	95 (50) _a_	2 (66.7)	2 (50) _ab_	50 (80.6) _b_	12 (50) _ab_	13 (40.6) _a_	1 (100)	10 (66.7) _ab_	
	Glass ionomer	65 (34.2)	2 (66.7) _ab_	0 (0)	16 (25.8)	3 (12.5)	11 (34.4)	0 (0)	7 (46.7)	
	MTA	29 (15.3) _a_	0 (0)	3 (75) _bcd_	18 (29) _ab_	8 (33.3) _abc_	21 (65.6) _c_	0 (0)	2 (13.3) _ad_	
	Biodentine	10 (5.3)	0 (0)	1 (25)	7 (11.3)	3 (12.5)	3 (9.4)	0 (0)	0 (0)	

Chi-square test, a–d: there is no difference between groups with the same letter in each row; frequency (percentage); multiple responses; oral and maxillofacial radiology was not included in this comparison; *p* < 0.05: There is a statistically significant difference.

**Table 5:** The distribution of answers to the question “Do you use any pulp coating agents for indirect pulp capping treatment?” according to place of work.

		Private	Public	University	Other/Private Hospitals	*p*
		***n* (%)**	***n* (%)**	***n* (%)**	***n* (%)**	**<0.001**
Do you use any pulp coating agents in indirect pulp capping treatment?	No	1 (2.1)	2 (2.2)	3 (2.4)	2 (3)	
	Ca(OH)_2_	29 (60.4) _a_	76 (85.4) _b_	101 (80.2) _b_	35 (53) _a_	
	Theracal LC	30 (62.5) _a_	29 (32.6) _b_	74 (58.7) _a_	50 (75.8) _a_	
	Glass ionomer	15 (31.3)	28 (31.5)	38 (30.2)	21 (31.8)	
	MTA	13 (27.1) _ab_	11 (12.4) _a_	37 (29.4) _b_	19 (28.8) _ab_	
	Biodentine	5 (10.4)	3 (3.4)	12 (9.5)	3 (4.5)	

Chi-square test, a, b there is no difference between groups with the same letter in each row; *n*: frequency; %: percentage; multiple responses; *p* < 0.05: There is a statistically significant difference.

**Table 6:** The distribution of answers to the question “Which permanent restoration material do you use after indirect pulp capping treatment?” according to place of work.

		Private	Public	University	Other Institutions	*p*
		***n* (%)**	***n* (%)**	***n* (%)**	***n* (%)**	
Which permanent restoration material do you use after indirect pulp capping treatment?	Composite	48 (100)	84 (94.4)	123 (97.6)	66 (100)	**<0.001**
	Compomer	3 (6.3)	5 (5.6)	15 (11.9)	7 (10.6)	
	Glass ionomer	7 (14.6)	12 (13.5)	24 (19)	5 (7.6)	
	Amalgam	0 (0)	45 (50.6) _a_	8 (6.3) _b_	2 (3) _b_	

Chi-square test, a, b: there is no difference between groups with the same letter in each row; *n*: frequency; %: percentage; multiple responses; *p* < 0.05: There is a statistically significant difference.

## Data Availability

The data is provided within the manuscript.
